# Intracellular lymphocyte protein biomarkers for early radiological triage in the human population

**DOI:** 10.1371/journal.pone.0331230

**Published:** 2025-09-09

**Authors:** Leah Nemzow, Alec Aljian, Thomas Boehringer, Michelle A. Phillippi, Maria Taveras, Eric Wang, Igor Shuryak, Lee A. Polikoff, Helen C. Turner

**Affiliations:** 1 Center for Radiological Research, Columbia University Irving Medical Center, New York, New York, United States of America; 2 Pediatric Critical Care Medicine, Warren Alpert School of Medicine, Brown University, Providence, Rhode Island, United States of America; University of Johannesburg Faculty of Health Sciences, SOUTH AFRICA

## Abstract

In the event of a large-scale radiological or nuclear emergency, a rapid, high-throughput screening tool will be essential for efficient triage of potentially exposed individuals, optimizing scarce medical resources and ensuring timely care. The objective of this work was to characterize the effects of age and sex on two intracellular lymphocyte protein biomarkers, BAX and p53, for early radiation exposure classification in the human population, using an imaging flow cytometry-based platform for rapid biomarker quantification in whole blood samples. Peripheral blood samples from male and female donors, across three adult age groups (young adult, middle-aged, senior) and a juvenile cohort, were X-irradiated (0–5 Gy), and biomarker expression was quantified at two- and three-days post-exposure. Mixed-effects modeling and ensemble machine learning approaches were employed to evaluate the influence of Age and Sex on biomarker expression and develop predictive models for radiation exposure classification. Although some Age and Sex effects on biomarker expression levels were observed when the data was stratified by targeted conditions of biomarker, day, age group, and sex, these variables were ultimately not retained as significant predictors of exposure classification. A single ensemble model successfully classified radiation exposure across all tested cohorts, with ROC AUC values ranging from 0.85 to 0.95 at the 1 Gy threshold and 0.81 to 0.87 at the 2 Gy threshold, high sensitivity values (91–96%) and low false-negative rates across all classifications. These findings support the use of BAX and p53 biomarkers in a blood test for efficient triage in large-scale emergencies, excluding individuals below exposure thresholds from unnecessary medical care with minimal risk of denying care to those truly exposed.

## Introduction

A large-scale radiological/nuclear (R/N) emergency may potentially expose hundreds of thousands or millions of individuals to levels of ionizing radiation that may cause acute health effects and necessitate urgent medical treatment. In such a scenario, it will be necessary to perform rapid, high-throughput screening to efficiently triage potentially exposed individuals for appropriate medical care and manage scarce medical resources [1–6]. Peripheral blood cells are easily accessible for sampling and highly sensitive to early radiation damage, serving as a viable target for radiation exposure screening [7]. The hematopoietic acute radiation syndrome (H-ARS) begins to manifest at absorbed doses of approximately 1 Gy [8,9], whereas casualties who receive doses > 2 Gy are at risk of developing more life-threatening symptoms of ARS, and potentially death within weeks if untreated [10]. While modeling studies as well as historical events across various R/N scenarios show varying magnitudes of casualties, similar patterns of dose exposure distributions are demonstrated with most individuals in the 0–1 Gy range [11–13], well below the generally proposed dose threshold of 2 Gy for administration of medical treatment in large-scale emergencies [4,14–18]. Thus, the development of triage tools that quickly and accurately identify these concerned citizens who do not require urgent medical care presents an impactful and critical step of the efficient emergency response. For this purpose, we have identified a panel of radio-responsive intracellular protein biomarkers [19] and developed an imaging flow cytometry (IFC)-based platform for rapid, high-throughput intracellular protein biomarker quantification within a whole blood sample [20–23].

Large-scale and widespread R/N incidents may expose the general population which is comprised of heterogeneous subpopulations. The development of bioassays for accurate exposure classification across all subpopulations can be challenging because several biological confounding factors such age, sex, race, comorbidities, pre-existing health conditions or infections, and genetic differences can exist, contributing to interindividual variability in radiosensitivity [24–26]. Underrepresentation of demographic subpopulations in study designs can result in overgeneralization of biomarker dose-response characteristics and lead to exposure classifications with lower sensitivity, specificity, and accuracy. Thus, there is a critical need to validate biomarker performance across demographic cohorts and systematically determine whether these confounding factors affect accurate exposure classification.

Age and sex are the largest and most basic characteristics for cohort stratification and may significantly influence biological outcomes yet historically have been neglected in experimental designs in many biomedical disciplines, including radiation research [27,28]. More recent studies have importantly addressed these characteristics, and age and sex are now well-known to affect the radiation response in various capacities, including molecular mechanisms and biological outcomes of carcinogenesis and mortality. Across the human and animal populations, radiation response has been shown to vary with age, as children, with rapidly dividing cells and developing tissues, and the elderly, with age-related deficiencies in DNA damage repair and response mechanisms, are particularly radiosensitive [29–40]. Sex-related differences in radiation responses have also been observed in both animal and human studies, and may be attributed to factors such as hormonal influences, X-linked genetics, and variations in DNA repair mechanisms [41–47]. In C57BL/6J mice, females show lower threshold doses for morbidity and mortality (~12 Gy vs. ~ 14 Gy in males) [48] and reduced survival times [49]. Human data suggest higher rates of long-term radiation-induced health risks in females, including carcinogenesis, [50] and sex-specific differences in radiation responses have led to recommendations for considering sex-specific approaches in radiotherapy for cancer treatment regimens [51]. Studies show that sex has been shown to affect radio-responsive gene expression patterns in human [52–54] and NHP blood [55], and in mouse models, sex-related variations have been observed across genomic, proteomic, metabolomic, and microRNA markers of radiosensitivity [24,56–65]. Further, age [66] and sex [67] have both been observed to affect expression of plasma protein radiation biomarkers in proteomic profiling studies conducted in C57BL/6 mice and have suggested the need for age- and sex-specific biomarker screening approaches and biodosimetry algorithms in these models.

The objective of this work was to characterize the effect of age and sex on radiation-induced responses of two previously identified lymphocyte intracellular protein biomarkers, BAX and p53 [19,20] for early classification of radiation exposure across the human population. These biomarkers are known to be involved in radiation-induced cellular responses such as apoptosis, DNA repair, and senescence, with their expression typically upregulated following exposure to ionizing irradiation. BAX (Bcl-2-associated X protein) is a pro-apoptotic protein with a well-established role in mediating radiation-induced cell death resulting from DNA damage [68–70]. Phosphorylation of the tumor suppressor protein p53 at serine 37 (Ser37) plays a role in the transcriptional regulation of the p53 protein in response to DNA damage [71]. In the present work, we collected peripheral blood samples from male and female adult donors across three adult age categories (young adult, middle-aged, seniors), as well as a juvenile cohort, and irradiated blood samples ex vivo with X rays up to 5 Gy. We used our previously developed human blood ex vivo culture model and high throughput imaging flow cytometry (IFC) platform [72] to rapidly quantify the radiation-induced increase in BAX and p53 protein expression two and three days after exposure and generate dose response curves for each donor. We evaluated the effect of Age and Sex on dose-dependent biomarker expression levels by mixed-effects and multiple variable regression modeling. We used a customized machine learning (ML) analysis pipeline to test the importance of all predictor variables, including Age and Sex, and train and test novel ensemble machine learning models for robust predictions of radiation exposure classifications at thresholds of 1 and 2 Gy.

## Methods

### Human blood sampling and irradiation

Peripheral blood samples from all donors were collected by venipuncture in BD Vacutainers® with Sodium-Heparin (BD Biosciences 367878). Two donor sets were recruited, (as seen in S1 Fig):

1) Adult donor set: Apparently healthy adult human volunteers were recruited with written consent obtained at the Columbia University Irving Medical Center (CUIMC) using approved IRB protocol AAAS7621. All blood donors reported no prior medical radiation exposure (dental X-rays allowed) in the past 6 months. Fresh blood samples were obtained from a total of 102 unique adult human donors (50 females and 52 males aged 22–82 years old, recruited between September 12^th^, 2022, and May 21^st^, 2024). An approximately equal numbers of male and female donors were recruited across 3 healthy adult age cohorts: 16 male and 16 female young adults (aged 18–30 years), 19 male and 19 female middle-aged adults (aged 31–59 years), and 17 male and 15 female seniors (aged 60 + years).
2) Juvenile donor set: Juvenile donors were recruited with obtained written consent from parents or guardians at the Hasbro Children’s Hospital emergency department (ED) at Brown University in Rhode Island using approved Lifespan IRB CMTT/PROJ Protocol 407721. Juvenile donors were generally admitted to the hospital for reasons relating to acute inflammatory conditions, such as appendicitis, upper respiratory infections, viral gastroenteritis, asthma exacerbation and abdominal pain. Blood samples were collected from a total of 30 unique donors (17 males and 13 females aged 3–17 years old; recruited between March 7^th^, 2022 and December 20^th^, 2022, and between March 18^th^ 2024 and October 29^th^ 2024), and shipped overnight at ambient temperature in CredoCubes™ (Peli BioThermal™, Series 22 15°C to 25°C). At the time of these juvenile recruitments, blood samples were also collected from 30 unique adult donors (12 males and 18 females aged 22–63 years old) at CUIMC and left overnight at ambient temperature in CredoCubes™.

All blood samples were irradiated with X-rays, as described in our previous work [72,73]. Briefly, blood samples from each donor were aliquoted (1–2 ml per dose) and mock irradiated or X-irradiated by X-RAD 320 biological irradiator (Precision X-Ray Inc., North Branford, CT) up to total doses of 1, 2, 3, 4, or 5 Gy, with the following conditions: custom Thoreaus filter (1.5 mm Al 0.25 mm Cu 1.25 mm Sn), 320 kVp, 12.5 mA, FSD 40, 0.95–98 Gy/min. Dose rate was validated with an ion chamber (Radcal® 10x6-6) before each sample irradiation.

### Sample preparation: culturing, staining, and fixation

Mock or X-irradiated peripheral whole blood samples were cultured and stained as previously described [72,73]. Briefly, 100 µL blood sample aliquots with 900 µL complete RPMI (with 15% FBS, 1% Pen-Strep added) were prepared in duplicates in Matrix™ 1.0 mL tubes (Thermo Fisher Scientific™ 3740TS, Waltham, MA) and cultured at 37°C, 5% CO_2_, for 2 or 3 days. After removing media supernatant, blood sample aliquots were surface stained for 20 minutes in the dark at room temperature with CD3-PE/Cy5 (mouse, monoclonal-HIT3a, BioLegend 300309, diluted 1:50) and CD20-PE/Dazzle™ 594 (mouse, monoclonal-2H7, BioLegend 302347, diluted 1:50) antibodies, lysed for 10 minutes at room temperature with eBioscience™ 1X RBC Lysis Buffer (Invitrogen, 00-4333-57), and then fixed at 4°C using the Cytofix/Cytoperm™ Fixation/Permeabilization Solution Kit (BD Biosciences, 554714, Franklin Lakes, NJ). Fixed leukocyte cells were washed with Perm/Wash™ buffer from the Cytofix/Cytoperm™ Fixation/Permeabilization Solution Kit and then one sample set was intracellularly stained with conjugated BAX-AlexaFluor488 (mouse, monoclonal-2D2, BioLegend 633604, diluted 1:200) antibody, and the other sample set was stained intracellularly with unconjugated phosphor-p53 Ser37 (rabbit, polyclonal, Cell Signaling Technology #9289, diluted 1:200) antibody for 1 hour in the dark, at room temperature, followed by a wash with Perm/Wash™ buffer. The p53 sample set was then stained with Alexa Fluor™ 488 (goat anti-rabbit, Invitrogen A11034, diluted 1:1000) for 1 hour in the dark, at room temperature. All samples were then washed twice with PBS, resuspended in 1 mL PBS, and stored at 4°C until sample acquisition.

### Imaging flow cytometry (IFC)

#### Sample acquisition.

Samples were acquired and analyzed by IFC similarly to previously described [72,73]. Briefly, sample aliquots were centrifuged and concentrated to a volume of 50 µl by removing excess PBS volume, and then acquired on the ImageStream MkII Imaging Flow Cytometer (Cytek, formerly Luminex Corporation, Austin, TX) with 488 nm excitation laser set to 200 mW, 785 nm excitation laser set to 1 mW (for side scatter, SSC) and a 40x brightfield (BF) magnification. Compensation samples stained with single fluorescence only were captured using the same laser settings as all other samples, but with BF and SSC inactivated. Focused and single cells were selected by gating, and 3,000 of these events were collected.

#### Analysis: Population selection and biomarker quantification.

The uniform analysis template that we previously created using Image Data Exploration and Analysis Software (IDEAS®, Cytek/Luminex ver. 6.2) was used to select for leukocyte populations and define regions of biomarker measurements [72,73]. Focused cells were selected by visually inspecting captured cell images and using the BF gradient root mean square (“Gradient RMS”) feature. Single cells were gated using a bivariate plot of BF area versus BF aspect ratio, eliminating debris and doublets. Lymphocyte populations were selected by morphological gating using a bivariate plot of BF area versus intensity of the SSC. Discrete event clusters with less BF area and SSC intensity were defined as “lymphocytes”. Morphological gating was validated by visualizing CD20+ and CD3 + events within the gated coordinates.

To quantify non-specific background signal, fluorescence intensity on the 480–560 nm detector (Channel 2) was examined in several unstained samples a mean “background” upper region boundary was set in the gated lymphocyte population. All intensity values above this “background” upper region boundary were defined as “Positive”. To measure biomarker expression in the gated lymphocyte populations, the uniform analysis template was applied to each biomarker-stained sample and automatically batch processed in IDEAS®. The percentage of single cells that appeared in the “Positive” region (“% Positive”) of the biomarker fluorescence intensity were computed for each acquired sampled.

#### Statistical analyses.

The following statistical analyses were performed using GraphPad Prism (version 10.3.0; GraphPad Software, Inc., La Jolla, CA). Normal distribution of all datasets was assessed by the Shapiro-Wilk normality test. Pearson’s product-moment correlation coefficients were computed to assess the relationship between dose and mean levels of biomarker expression. The differences of mean BAX and p53 expression between Age and Sex cohorts on Days 2 and 3 post 0–5 Gy X-irradiation were analyzed by repeated-measures mixed-effects modeling (using a compound symmetry covariance matrix and fit using Restricted Maximum Likelihood), followed by Šidák’s multiple comparison test (tests differences between Age or Sex cohorts at each dose independently while controlling for family-wise error rate; alpha threshold level set to 0.05). For all statistical analyses, two-tailed p values less than 0.05 were considered statistically significant.

Multivariable linear regressions with main effects and multiplicative three-way interactions between Age, Sex, Dose on each Day were performed using *R* (version 4.4.1). Interaction terms were tested for significance to determine how predictors jointly influence biomarker expression on Day 2 and Day 3. Significance testing was conducted using p-values from t-tests for each coefficient, with p < 0.05 considered significant.

#### Machine learning analyses.

The data from all experiments were compiled in the R 4.4.1 programming language [74]. Missing values for the biomarkers were input using the MissForest algorithm in R. The complete data sets can be found in S1 Table (adult and juvenile). The main variables in the adult data set were:

- Time after irradiation (Day 2 or 3)
- Sex (female = 0, male = 1)
- Age (“young adult” = 0, “middle-aged” = 1, “senior” = 2)
- Age (as a continuous variable)
- BAX
- p53

Exposure Index (0 = 0 Gy, 1 = all doses > 0 Gy), and Intervention Index (0 = 0–1 Gy; 1 = all doses > 2 Gy) were treated as the target variables to be predicted by the ML models, using the other variables as predictors.

The main variables in the juvenile data set were:

- Sex (female = 0, male = 1)
- BAX
- p53

Due to the fewer number of tested doses in the juvenile dataset, only the Exposure Index (0 = 0 Gy, 1 = all doses > 0 Gy) was treated as the target variable to be predicted by the ML models, using the other variables as predictors.

The ML analyses were performed in Python 3.10.4, Jupyter (https://jupyter.org/). Data were split randomly into training and testing parts (50% each). First, feature selection was performed using the Boruta algorithm (implemented by the boruta_py Python package [75]) to iteratively compare the importance of each predictor (original feature) compared to shadow features (randomly shuffled copies of original features) using Random Forest (RF). Performance criteria were set to ≥ 100^th^ percentile of the randomized “shadow” features, using α = 0.05 with Bonferroni correction as the statistical threshold.

The retained predictors (which passed the Boruta screening) were used to train ensemble stacked ML algorithms to classify samples by Exposure Index (0 = 0 Gy; 1 = all doses > 0) and Intervention Index (0 = 0–1 Gy; 1 = 2–5 Gy). During stacking, several ML methods (level 0 models, which here were logistic regression, CatBoost, XGBoost, Light GBM, Random Forest (RF), K-Nearest Neighbors, Gaussian Naïve Bayes, and Support Vector Machines) were applied to the training data with repeated k-fold cross-validation (10-fold, repeated 10 times), as follows: Predictions of each level 0 model on data folds that were withheld during cross validation were recorded. These predictions served as inputs to train meta-models (level 1) which learned how to best combine the predictions of the level 0 models to predict the target variables. In this case, the best level 1 meta-model for predictions of both classification indices was Logistic Regression, based on comparing the mean performance scores (balanced accuracy metric) for all models on the repeated cross validations. The reserved testing data was then applied to the ensemble meta-models to test the novel algorithms’ predictive performances using unseen data.

## Results

### Radiation dose-dependent biomarker expression

**Fig 1:**
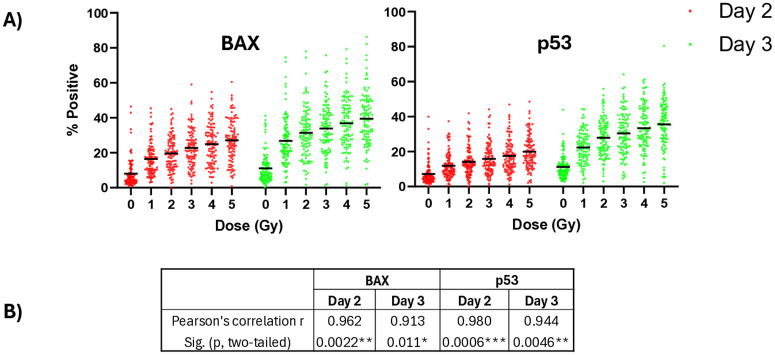
Radiation dose-dependent biomarker expression. A) Data represent BAX and p53% Positive values in all adult donors. Black bars represent the mean % Positive biomarker value measured at each X-ray dose; N = 101–102 total donor samples tested at each dose. Descriptive statistics of mean, fold change increases between each dose point, and standard deviations can be found in **Table 1**. B) Pearson’s product-moment correlations were computed to evaluate the strength of association between mean levels of biomarker expression and dose. p values represent significance of Pearson’s correlation (*p < 0.5, **p < 0.01, ***p < 0.001).

**Fig 1A** shows dose-dependent increases in mean expression of both BAX and p53 biomarkers on Days 2 and 3 across all adult donors (101–102 donors per each dose). Pearson’s product-moment correlation coefficients demonstrate strong, positive correlations between mean biomarker expression values and X-ray doses across both days (**Fig 1B**; r = 0.91–0.98; p < 0.05 – < 0.001). Further evaluation of the biomarker means as a function of dose shows BAX and p53 expression levels are more amplified on Day 3 as opposed to Day 2, with BAX and p53 baseline values (0 Gy) more elevated on Day 3 by 33% and 56%, respectively, and simple regression slopes across all the doses significantly higher on Day 3 (p values ranging < 0.001–0.0001). On both days, the largest mean increase per dose is observed between 0 Gy and 1 Gy with fold changes for BAX of 2.03 on Day 2 and 2.43 on Day 3, and fold changes for p53 of 1.66 on Day 2 and 1.99 on Day 3, followed by smaller fold changes between tested doses in the 1–5 Gy range (**Table 1**). Standard deviations (SD) show that the distribution of the biomarker values (% Positive) on each Day range from approximately 8% to 15% of the mean values at each dose, suggesting a need to identify factors that potentially contribute to this variability.

**Table 1:** Descriptive statistics of measured biomarker expression at each X-ray dose, as visually presented in Fig 1.

	BAX								p53							
	Day 2				Day 3				Day 2				Day 3			
	Mean	FC/dose	SD	N	Mean	FC/dose	SD	N	Mean	FC/dose	SD	N	Mean	FC/dose	SD	N
**0 Gy**	8.05		7.92	101.00	11.04		8.59	102.00	7.17		6.32	102.00	11.21		6.58	102.00
**1 Gy**	16.35	2.03	9.04	102.00	26.78	2.43	13.45	102.00	11.91	1.66	7.23	102.00	22.32	1.99	9.04	101.00
**2 Gy**	19.65	1.20	9.63	102.00	31.37	1.17	14.08	102.00	14.09	1.18	8.09	101.00	27.91	1.25	10.68	102.00
**3 Gy**	22.69	1.15	10.52	102.00	33.91	1.08	13.88	102.00	15.81	1.12	8.64	102.00	30.36	1.09	12.23	102.00
**4 Gy**	24.91	1.10	11.28	102.00	36.83	1.09	14.52	102.00	17.50	1.11	9.15	102.00	33.41	1.10	12.69	102.00
**5 Gy**	27.09	1.09	12.03	102.00	39.42	1.07	15.50	102.00	19.95	1.14	9.43	102.00	35.55	1.06	13.39	101.00

“FC (fold change)/dose” is calculated as the (mean value for each dose point/ mean value of the preceding dose point). “SD” = Standard Deviation.

### Effect of age on biomarker dose responses

**Fig 2:**
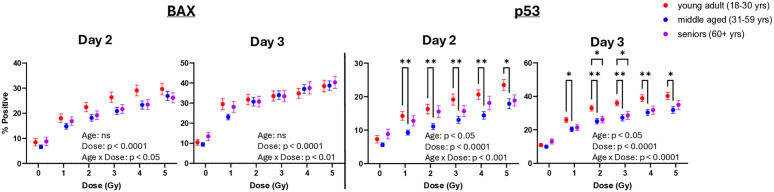
Effect of Age on biomarker dose-response levels. Data represent BAX and p53% positive values in all 102 adult donors stratified into Age recruitment cohorts as described in the Materials and Methods. n = 32 - 38 donor samples tested at each dose; error bars represent ± SEM. Mixed-effects modeling was performed to evaluate the effects of Dose and Age on mean levels of biomarker expression; complete results can be found in S2 Table. p values in the figure text represent significance of Age and Dose main effects on biomarker levels; “ns” = not significant. Asterisks denote significantly different mean values between Age groups at each Dose (Šidák’s post-hoc test); * p < 0.05, ** p < 0.01.

To examine the effects of Age and Dose on biomarker levels, we conducted repeated-measures mixed-effects analyses for these variables on Days 2 and 3. Main effect analyses in **Fig 2** show that Dose has a statistically significant effect on levels of both biomarkers on both days (p < 0.0001), whereas Age only has significant effect on p53 expression levels on both days (p < 0.05). However, interactions between Age categories and Dose reached statistical significance for both BAX and p53 on both days (p < 0.05–0.0001). To evaluate targeted differences between Age cohorts at each Dose, we performed Šidák’s Post Hoc multiple comparisons. Results show that the mean values of p53 levels measured in the young adult cohort were significantly higher than the middle-aged at all X-ray doses (1–5 Gy) on Days 2 and 3, and higher than the seniors on Day 3. Overall, these findings suggest that Age directly influences p53 biomarker variance but not BAX, while also modifying the dose-response relationship for both BAX and p53, with the young adult cohort exhibiting a stronger response.

### Effect of sex on biomarker dose responses

**Fig 3:**
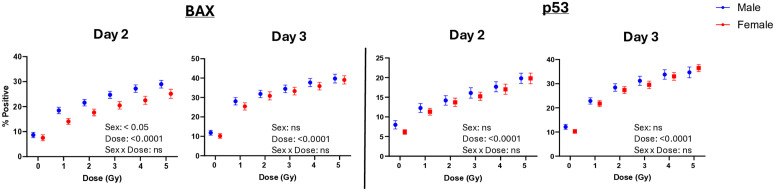
Effect of Sex on biomarker dose-response levels. Data represent BAX and p53 % Positive values in all adult 102 donors stratified by Sex recruitment cohorts as described in the Materials and Methods. n = 50–52 donor samples tested at each dose; error bars represent ± SEM. Mixed-effects modeling was performed to evaluate the effects of Dose and Sex on mean levels of biomarker expression; complete results can be found in S2 Table. p values in the figure text represent significance of Sex and Dose main effects on biomarker levels; “ns” = not significant. Multiple comparisons found no significant differences between group means at each dose (Šidák’s post-hoc test).

To examine the effects of Sex and X-ray Dose on biomarker levels, we also performed repeated-measures mixed-effects analysis for these variables on Days 2 and 3 (**Fig 3**). Main effects analyses show that Dose has a statistically significant effect on levels of both biomarkers and on both days (p < 0.0001), and that Sex affects radiation-induced BAX expression only on Day 2, with males exhibiting higher biomarker levels. The effect of Sex and Dose variable interactions did not reach significance for either biomarker on either Day, indicating that biomarker dose responses are not influenced by Sex.

### Effect of Age x Sex interactions on biomarker dose responses

Here, we examined the combined effects of Age and Sex on the dose-response levels of each biomarker through multiple linear regression analyses, testing the three-way interactions between Age × Sex × Dose (**Table 2**). Our results reveal a significant three-way interaction in BAX on Day 3 (Age × Sex × Dose, p = 0.02933) with an estimated coefficient of −0.08189, indicating that the effect of Dose on BAX expression decreases with increasing Age in males. This suggests an attenuated dose-response relationship in older males compared to females, highlighting a combined Age- and Sex-dependent effect on dose response that was not observed through main effects or two-way interactions alone. However, no significant three-way interactions were found in other conditions, which reinforces that the significant main effects of Age and Sex, along with the two-way interactions between Sex × Dose and Age × Dose (as seen in mixed-effects analyses in **Figs 2** and **3**), do not depend on further interactions between Age and Sex.

**Table 2:** Effect of age x sex interactions on dose responses.

BAX						p53					
Day 2			Day 3			Day 2			Day 3		
Estimate	Std. Error	p value	Estimate	Std. Error	p value	Estimate	Std. Error	p value	Estimate	Std. Error	p value
−0.04213	0.02731	0.123445	−0.08189	0.03749	0.02933 *	−0.02181	0.02241	0.3308	−0.00845	0.030371	0.7811

Multiple variable regressions were performed to test the multiplicative effect of Age, Sex, and Dose on BAX and p53 biomarker levels. For the 3-way Age:Sex:Dose interactions, estimated coefficients (“Estimate”), standard error (Std. Error) and p values are presented.

### Distribution of biomarker expression distribution across Age and Sex cohorts

It is worth noting that while some targeted effects of Age, Sex, Age x Dose, Sex x Dose, and Age x Sex x Dose on biomarker expression levels reach statistical significance in some conditions, even in those cases, examination of the data’s distribution show that the interquartile ranges between groups still overlap by at least 50% (S2 and S3 Figs). This suggests that the central tendencies of biomarker levels between Age or Sex cohorts are somewhat similar, and that the full range of biomarker distributions at each dose are not fully explained by Age or Sex factors.

### Machine learning exposure classifications in the adult population

All the dose-response data from across all 102 adult donors collected 2- and 3-days post 0–5 Gy exposure were used to train and test ML ensemble models for predicting exposure classifications at 1 Gy and 2 Gy dose thresholds. Boruta feature selection discarded Age and Sex as important variables for predicting exposure classifications (across both days and target thresholds) and stacked ensemble models were trained and tested using the remaining variables of BAX and p53 expression levels (complete table of predicted values on the testing dataset can be found in S3 Table). **Table 3** shows the performance metrics for classifications generated from the testing data applied to the trained ensemble models. For exposure classifications at the 1 Gy threshold, the stacked model achieved an ROC AUC of 0.85 with 88% accuracy on Day 2 and a stronger ROC AUC of 0.95 with 92% accuracy on Day 3. When the data from Days 2 and 3 were combined, the ensemble model produced an ROC AUC of 0.90 with 85% accuracy. For exposure classifications at the 2 Gy threshold, the stacked model achieved an ROC AUC of 0.82 with 80% accuracy on Day 2, 0.87 with 80% accuracy on Day 3, and 0.81 with 75% accuracy when the data from Days 2 and 3 were combined. Specificity values (true negatives/ true negatives + false positives) across both dose thresholds on all days range from 46–67% due to the relatively high numbers of false positives relative to those predicted as unexposed. However, sensitivity values (true positives/ true positives + false negatives) range from 91–96%, highlighting the very low numbers of false negatives relative to those predicted as exposed.

**Table 3:** Machine learning predictions of exposure indices.

	1 Gy threshold				2 Gy threshold			
Days	AUC	Accuracy	Sensitivity	Specificity	AUC	Accuracy	Sensitivity	Specificity
**2 + 3**	0.90	0.86	0.95	0.46	0.81	0.75	0.91	0.47
**2**	0.85	0.88	0.96	0.46	0.82	0.80	0.93	0.52
**3**	0.95	0.92	0.96	0.67	0.87	0.80	0.93	0.52

The “1 Gy threshold” column presents ROC curve discrimination of the testing data between unexposed (0 Gy) and exposed (1–5 Gy) samples; the “2 threshold” column presents ROC curve discrimination of the testing data between no intervention recommended (0 and 1 Gy) and intervention recommended (2–5 Gy). AUC = area under the curve. Full testing dataset and Exposure Index predictions can be found in S3 Table.

### Biomarker dose responses in the juvenile population

**Fig 4:**
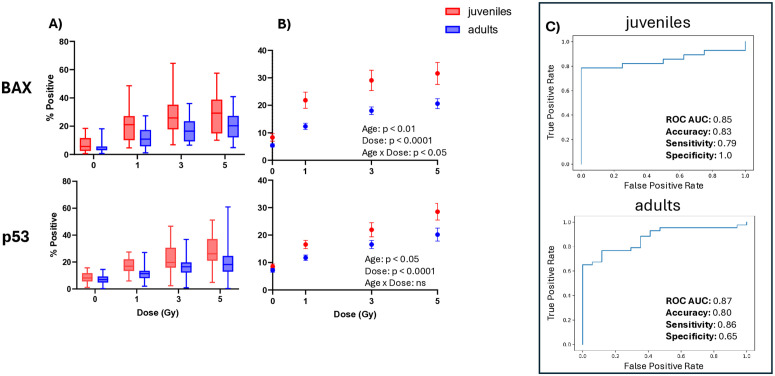
Biomarker dose-responses in juvenile population. Data represent BAX and p53% positive values in all “overnight” donors, stratified by Age group/sample source; n = 18 total juvenile donor samples tested at each dose, n = 30 total adult donor samples tested at each dose**. A) Distribution of biomarker dose-response level in adult and juvenile cohorts.** Within each box, horizontal dark colored lines represent median values; boxes extend from the 25th to the 75th percentile of each group’s distribution of values; “whiskers” (vertical lines extending above and below each box) represent adjacent values (i.e., the most extreme values within 1.5 interquartile range of the 25th and 75th percentile of each group); dots denote observations outside the range of adjacent values (2.5–97.5%). B) **Effect of juvenile age on biomarker dose-response levels.** Error bars represent ± SEM. Mixed-effects modeling was performed to evaluate the effect of Dose and Age on mean levels of biomarker expression; complete results can be found in S4 Table. p values in the figure text represent significance of Sex and Dose main effects on levels; “ns” = not significant. C) **Machine Learning predictions of Exposure Index.** Performance metrics for discrimination of the testing data between unexposed (0 Gy) and exposed (1, 3, 5 Gy) samples are presented; full testing dataset and Exposure Index predictions can be found in S5 Table.

To further evaluate the effect of Age on biomarker expression in this demographic study, we also examined blood samples collected and shipped from a juvenile (aged 3–17 years) cohort (paired with healthy adult blood stored overnight to mimic shipping time) that were cultured for 2 days following X-irradiation. Interestingly, low lymphocyte counts (< 500) at the 0 Gy baselines were found in 36% of recruited juvenile donor samples and were excluded from the dataset due to insufficient number of target cells for statistically robust and reliable biomarker quantification. Low lymphocyte counts were not observed in the paired adult samples. Analysis of biomarker expression levels across all unirradiated and irradiated doses in each Age cohort reveals higher median values in the juvenile cohort, as well as wider interquartile and overall ranges (**Fig 4A**). Mixed-effects analyses performed on the juvenile and adult cohorts show that for both BAX and p53 biomarkers, the main effects of Age and Dose were highly significant (p < 0.001 and < 0.0001, respectively), while the interactions of Age x Dose only reached significance for BAX (p < 0.05) (**Fig 4B**). Šidák’s Post Hoc multiple comparisons between mean BAX biomarker measurements in the juvenile and adult cohorts at each Dose did not detect significant differences (potentially due to unequal sample sizes and high variability). However, the data show a late divergence between the Age cohorts, with a small baseline difference of 2.2% in unirradiated samples that increases to ~8–9% across all irradiated doses, likely contributing to the significant Age x Dose interaction. These results suggest that while both Age and Dose independently affect biomarker expression, dose responses in the juvenile cohort are more pronounced for BAX. We utilized the ML pipeline to determine whether exposure status may be predicted based on BAX and p53 expression at the 1 Gy threshold within a juvenile population. Discrimination between unexposed (0 Gy) and exposed (1, 3, 5 Gy) status in the juvenile cohort yielded an ROC AUC of 0.85 with 83% accuracy, performing similarly to the paired adult cohort in this dataset with an ROC AUC of 0.87 with 80% accuracy on Day 2 after exposure (**Fig 4C**).

## Discussion

An R/N incident may occur at any time or place and potentially expose hundreds of thousands or even millions of individuals to levels of ionizing radiation that can cause detrimental health effects. As medical resources are likely to be limited during an emergency scenario, there exists a critical need to develop a bioassay that enables early and accurate identification of individuals at risk of radiation-induced adverse health effects, effectively distinguishing them from those who are not. Given the widespread nature of R/N events that can expose the general civilian population, the bioassay must consider potential variations in radiosensitivity across human demographic factors and individual biological responses. Here in this work, we used the human blood ex vivo model to generate dose-response curves for two radio-responsive intracellular lymphocyte biomarkers (BAX and p53) across 102 adult donors with approximately equally sized cohorts of Sex (male and female) and Age (young adult, middle-aged, senior) on days 2 and 3 after exposure. We evaluated the effects of Age (**Fig 2**) and Sex (**Fig 3**) on biomarker dose responses and successfully used the collective datasets to generate ensemble ML models for early classifications of radiation across all Sex and Age cohorts within three days post-exposure (**Table 2**). While some targeted Age and Sex effects on biomarker variance across doses were seen, Boruta feature selection rejected Age and Sex as important predictors, suggesting that a universal algorithm could be used across these populations. The results presented here from this focused demographic study serve as an important step towards the development of a high-throughput blood assay for medical triage of the general population following a large-scale R/N incident.

In this work, the distribution of biomarker values across all the healthy adult heterogenous subpopulations show appreciable interindividual variability and distribution range in both baseline (0 Gy) and radiation dose-response (1–5 Gy) levels (**Fig 1** and **Table 1**). However, when the data was stratified by Age or Sex (S2 and S3 Figs), much of the wide range of distribution remains, suggesting that the full variance in the data is only partially explained by Age and Sex effects. Further investigation is needed to identify other potential confounding factors, such as genetic composition, pre-existing conditions, underlying biological factors (e.g., obesity), and lifestyle differences (e.g., smoking, alcohol consumption), that may contribute to interindividual variability in biomarker levels.

Despite the presence of interindividual variability and targeted Age and Sex effects within this large adult demographic dataset, these data were successfully used to train and test ML-based ensemble models for classifying radiation exposure at two dose thresholds, 1 and 2 Gy (**Table 3**). Strong discrimination of exposure at the 1 Gy threshold has been achieved with ROC AUC values across the tested time points ranging 0.85–0.95 and accuracies ranging 0.86–0.92 across all Sex and Age cohorts. As H-ARS symptoms could begin as low as 0.75 Gy [8,9], future work that includes doses < 1 Gy will determine the lowest detection range of these biomarkers. In a large-scale emergency scenario with scarce resources, 2 Gy is the generally established threshold for medical intervention and early triaging will aim to exclude individuals who do not meet this threshold [4,14–18]. We show here that discrimination of samples at the 2 Gy threshold yielded moderately strong results with ROC AUC values across the tested time points ranging 0.81–0.87, and accuracies ranging 0.75–0.80. Although these classifications did not attain very high specificity; classification results at both thresholds demonstrated exceptionally high sensitivity (91–96%). This indicates that while the bioassay produces some false positives, the very low false-negative rates provide strong reassurance that individuals identified as negative are truly below the exposure thresholds, with minimal risk of denying care to those who exceed the thresholds. Assay specificity and accuracy may be improved with future studies that test replicate samples to reduce technical or methodological variances that could contribute to perceived interindividual differences in biomarker expression, as well as by developing improved methods of sample normalization. Overall, the high ROC AUC and sensitivity values support the use of BAX and p53 intracellular lymphocyte protein biomarkers in developing a reliable exclusion test that is robust against interindividual variability within the general adult population.

Juveniles could represent a vulnerable population in an R/N incident, and it is critical that developed bioassays for accurate reconstruction of radiation exposure are inclusive of this population. We importantly also validated the performance of BAX and p53 lymphocyte protein biomarkers in the juvenile population two days after radiation exposure, using shipped blood samples from patients admitted to a pediatric ED. The data show that BAX and p53 baseline values and distribution ranges were higher in the juvenile cohort compared to the paired apparently healthy adults, respectively (**Figs 4A** and **4B**). Further studies with healthy juvenile donors are needed to determine the extent that this phenomenon is characteristic of the juvenile age or due to potentially confounding inflammatory conditions present in the blood samples acquired from the pediatric ED. Despite these differences, ML-based exposure classification was generally successful (**Fig 4C**), supporting the use of these biomarkers in a universal bioassay for triaging exposed victims in both juvenile and adult populations. Future tests with larger juvenile sample sizes will determine whether juvenile and adult exposure-predictive ML algorithms may be merged and demonstrate sufficient triaging capabilities across age cohorts. It is worth noting that low lymphocyte counts were observed in 36% of the acquired juvenile blood samples (and were consequently excluded from analysis), suggesting clinical lymphopenia or granular demargination (which may lower the proportion of circulating lymphocytes present in the blood sample) in these donors. This highlights the need to establish minimal thresholds of lymphocyte counts during sample collection to allow for reliable biomarker quantification across varied immunological profiles.

Human ex vivo blood culture models serve as flexible and practical platforms for biodosimetry biomarker discovery and detailed acute dose-response studies in the general human population. Due to ethical regulations, human in vivo radiation models are typically restricted to cancer patients undergoing radiotherapy, and the difficulties in obtaining these subjects, along with the potential confounding factors, they may have, limit the use of this model in a large population study. While useful as a preliminary approach for in vivo validation and longitudinal measurements of biomarker signatures, laboratory-based animal models are phylogenetically removed from humans and are also typically constrained to relatively small sample sizes and low genetic heterogeneity with only single strains, species, ages, or sexes being often included in the study designs. Human ex vivo models importantly allow for large sample numbers and study designs that are inclusive of many demographic subpopulations, which are critical factors in the successful development of accurate biodosimetry algorithms. However, ex vivo models may not accurately recapitulate all radiation-induced DNA damage or cell stress responses that occur in a true physiological context, and the transferability of the biomarker expression signature to a human in vivo model must be verified. Gene-based biomarker signatures have been successfully validated with matching expression patterns [76] and exposure classifications [77] in human ex vivo and in vivo models (patients being treated with total-body radiotherapy), supporting the use of the ex vivo platform to develop radiation dosimetric signatures that are relevant for in vivo exposures. Future work may similarly utilize a targeted group of radiotherapy patients to validate the transferability of these intracellular lymphocyte protein biomarkers for exposure classifications in a human in vivo context.

In this work, biomarker expression was quantified on an IFC as a laboratory-based bioassay for rapid exposure classification. In actualized emergency scenarios, laboratory-based triage tools would require transportation of peripheral blood samples to a centralized location, and the effects of shipping on biomarker expression and exposure classifications should be considered and further investigated. Transition to a point-of-care device that is deployable in the field may be desirable to obtain faster results and further alleviate the burden on laboratory resources, such as shipping networks and data transfer systems, which could already be taxed by gold standard bioassays required for definitive dose determinations (e.g., cytogenetic analysis).

The intracellular lymphocyte protein biomarkers, BAX and p53, show utility for rapid exposure classification and triaging in the work presented here, and these biomarkers may also be included in future efforts to develop a multiparametric bioassay for reconstruction of definitively absorbed doses. Integration of these protein biomarkers into a comprehensive panel alongside additional biomarkers with diverse dose-response dynamics and kinetics could further enhance the predictive accuracy of dose estimation within a wider dose range and provide more detailed treatment recommendations for clinicians. Additionally, the ML pipeline developed in our group is importantly adaptable for integrating diverse predictors from IFC and other sources, including proteins, genes, metabolites, and cell counts, into a multiparametric panel. Our ML-based integrative multiparametric approach has been successfully demonstrated [78,79], including in recent work in our laboratory using an in vivo C56BL/6 mouse model, which combined a panel of intracellular lymphocyte proteins and B and T cell counts for accurate dose reconstruction [21,22].

## Supporting information

S1 Table Complete set of biomarker values. Grey cells represent values that were input by the MissForest algorithm. The meaning of each variable is described in Materials and Methods section in the main text.
(XLSX)

S2 Table Repeated-measured mixed-effects modeling results for Dose and Age and Sex variables in the fresh adult dataset. (XLSX)

S3 Table Level 1 model predictions of Exposure Index based on the testing portion of the fresh adult dataset generated by the stacking ensemble. The meaning of each variable is described in Materials and Methods section in the main text.
(XLSX)

S4 Table Repeated-measured mixed-effects modeling results for Dose and Age variables in the juvenile shipped dataset. (XLSX)

S5 Table Level 1 model predictions of Exposure Index based on the testing portion of the juvenile shipped dataset generated by the stacking ensemble. The meaning of each variable is described in Materials and Methods section in the main text.
(XLSX)

S1 Fig Age and sex distribution of recruited participants in adult and juvenile donor sets. Bar chart displays the number of male (blue) and female (pink) participants recruited across age groups within two sets: (1) Adult donor set, stratified into age groups 18–30, 31–59, and 60 + ; and (2) Juvenile donor set, divided into age groups 3–17 and 22–63. The total number of male and female donors in each cohort is also indicated.
(TIF)

S2 Fig Effect of Age on biomarker dose-response level distributions. Data represent BAX and p53% Positive values in all 102 adult donors stratified into Age recruitment cohorts as described in the Materials and Methods. Within each box, horizontal dark colored lines represent median values; boxes extend from the 25th to the 75th percentile of each group’s distribution of values; “whiskers” (vertical lines extending above and below each box) represent adjacent values (i.e., the most extreme values within 1.5 interquartile range of the 25th and 75th percentile of each group); dots denote observations outside the range of adjacent values (5–95%); n = 32–38 donor samples tested at each dose.
(TIF)

S3 Fig Effect of Sex on biomarker dose-response level distributions. Data represent BAX and p53% Positive values all adult 102 donors stratified by Sex recruitment cohorts as described in the Materials and Methods. Within each box, horizontal dark colored lines represent median values; boxes extend from the 25th to the 75th percentile of each group’s distribution of values; “whiskers” (vertical lines extending above and below each box) represent adjacent values (i.e., the most extreme values within 1.5 interquartile range of the 25th and 75th percentile of each group); dots denote observations outside the range of adjacent values (2.5–97.5%); n = 50–52 donor samples tested at each dose.
(TIF)
