# Supplementary figures and images for: Intracellular lymphocyte protein biomarkers for early radiological triage in the human population
Source: PLoS One. 2025 Sep 9;20(9):e0331230. doi: 10.1371/journal.pone.0331230 (PMC12419610; doi:10.1371/journal.pone.0331230)

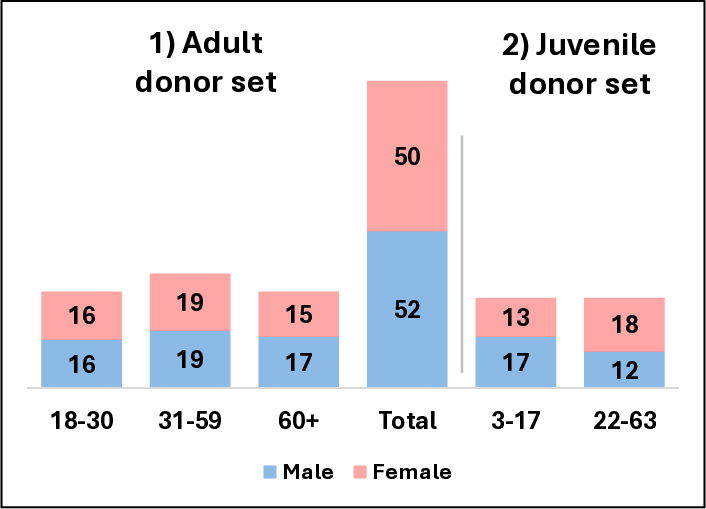

Supplement: S1 Fig — Bar chart displays the number of male (blue) and female (pink) participants recruited across age groups within two sets: (1) Adult donor set, stratified into age groups 18–30, 31–59, and 60 + ; and (2) Juvenile donor set, divided into age groups 3–17 and 22–63. The total number of male and female donors in each cohort is also indicated. (TIF) [file pone.0331230.s006.tif]

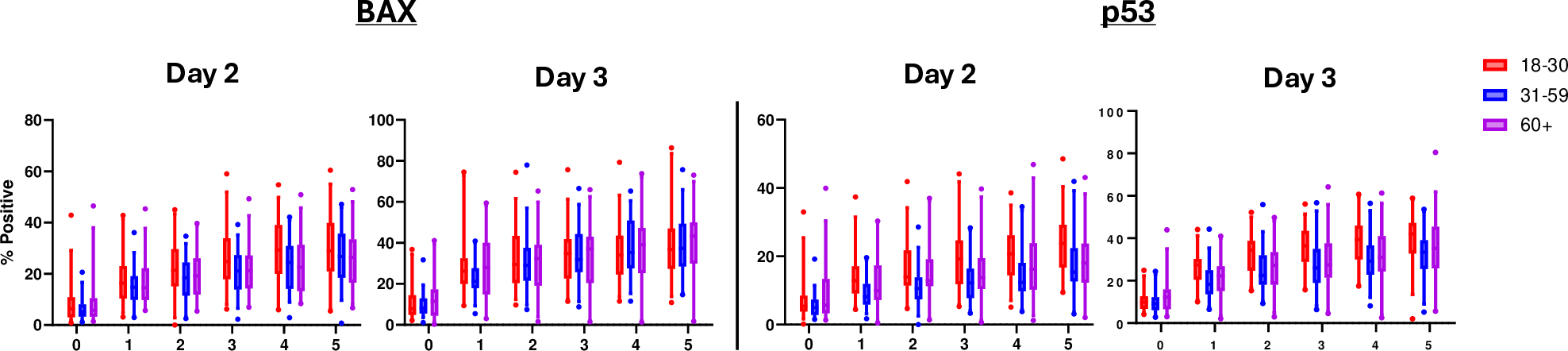

Supplement: S2 Fig — Data represent BAX and p53% Positive values in all 102 adult donors stratified into Age recruitment cohorts as described in the Materials and Methods. Within each box, horizontal dark colored lines represent median values; boxes extend from the 25th to the 75th percentile of each group’s distribution of values; “whiskers” (vertical lines extending above and below each box) represent adjacent values (i.e., the most extreme values within 1.5 interquartile range of the 25th and 75th percentile of each group); dots denote observations outside the range of adjacent values (5–95%); n = 32–38 donor samples tested at each dose. (TIF) [file pone.0331230.s007.tif]

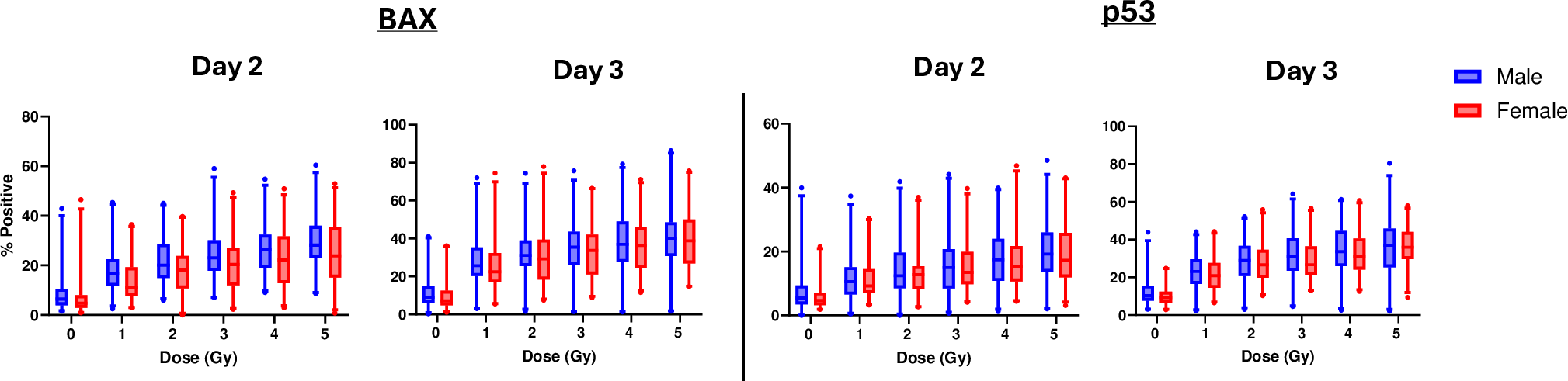

Supplement: S3 Fig — Data represent BAX and p53% Positive values all adult 102 donors stratified by Sex recruitment cohorts as described in the Materials and Methods. Within each box, horizontal dark colored lines represent median values; boxes extend from the 25th to the 75th percentile of each group’s distribution of values; “whiskers” (vertical lines extending above and below each box) represent adjacent values (i.e., the most extreme values within 1.5 interquartile range of the 25th and 75th percentile of each group); dots denote observations outside the range of adjacent values (2.5–97.5%); n = 50–52 donor samples tested at each dose. (TIF) [file pone.0331230.s008.tif]
